# Factors that influence the adoption of rehabilitation technologies: a multi-disciplinary qualitative exploration

**DOI:** 10.1186/s12984-023-01194-9

**Published:** 2023-06-20

**Authors:** Jessie Mitchell, Camila Shirota, Kelly Clanchy

**Affiliations:** 1 The Hopkins Centre, Menzies Health Institute Queensland, Griffith University, Brisbane, Australia; 2 School of Health Sciences and Social Work, Griffith University, Southport, Australia

**Keywords:** Rehabilitation technology, Qualitative analysis, Co-design, Clinical translation

## Abstract

**Background:**

Technological innovation is recognised as having the potential to enhance rehabilitation for people with disability. Yet, resistance to, and abandonment of, rehabilitation technology is prevalent and the successful translation of technology into rehabilitation settings remains limited. Therefore, the aim of this work was to develop an in-depth, multi-stakeholder perspective on what influences the adoption of rehabilitation technologies.

**Methods:**

Semi-structured focus groups were conducted as part of a larger research project aiming to facilitate the co-design of a novel neurorestorative technology. Focus group data were analysed using a five-phase hybrid deductive-inductive approach to qualitative data analysis.

**Results:**

Focus groups were attended by 43 stakeholders with expertise in one or more of the following fields: people with disability, allied health, human movement science, computer science, design, engineering, ethics, funding, marketing, business, product development, and research development. Six main themes influencing the adoption of technology in rehabilitation were identified: cost beyond the purchase price, benefits to *all* stakeholders, trust to be earned in technology, ease of technology operation, ability to access technology, and the ‘co’ in co-design. All six themes were found to be interrelated; in particular, the importance of direct stakeholder engagement in the development of rehabilitation technologies (the ‘co’ in co-design) was prevalent in all themes.

**Conclusions:**

A range of complex and interrelated factors influence the adoption of rehabilitation technologies. Importantly, many of the issues that have the potential to negatively impact rehabilitation technology adoption may be addressed during development by utilising the experience and expertise of stakeholders who influence its supply *and* demand. Our findings state that a wider cohort of stakeholders needs to be actively engaged in the development of rehabilitation technologies to better address the factors that contribute to technology underutilisation and abandonment and facilitate better outcomes for people with disability.

## Background

Global estimates suggest that 2.41 billion people live with conditions that would benefit from rehabilitation, with as many as one in every three people needing rehabilitation at some point due to illness or injury [1]. Technological innovation is widely recognised as having the potential to enhance rehabilitation offerings [2–6]. As a result, rehabilitation technologies have attracted significant attention and resources internationally. The World Health Organisation, for example, introduced the Global Cooperation on Assistive Health Technology in 2014, one of the focuses of which is to prioritise research and innovation in assistive technology [7]. However, the successful translation of technological innovations into rehabilitation settings remains limited. Only one in ten people are estimated to have access to the assistive products they require, an inequity that is even larger in low- and middle-income countries [8]. Further, abandonment of assistive technologies has been reported to average almost 20% [9, 10], and data suggests that resistance to the adoption of health technologies still exists among health service providers [11].

Reasons for technology resistance and abandonment are complex, and are largely centred around a mismatch between user needs and available solutions [12–14]. As a result, awareness has increased around the importance of user-centred approaches to the development of rehabilitation technology, with focus placed on end-user collaboration in designing, acquiring, testing, and evaluating technologies in real-world settings (i.e., beyond research facilities) [15, 16]. Efforts have been made to identify and address barriers to rehabilitation technology adoption using the perspectives of people with a disability and health service providers [17, 18]. Yet, a review of published literature suggests that many of the rehabilitation technologies being studied still do not align with the priorities of the people with disability who they are designed for, or the preferences of the health services providers who are expected to deliver them [19].

Further, while these end-user perspectives are undeniably crucial, a number of other stakeholders also influence the supply and demand of rehabilitation technologies. Rehabilitation technology development and implementation occur within complex multi-level social systems [20, 21]. Other stakeholders relevant to the development and delivery of rehabilitation technology include, but are not limited to, carers of people with disability, administrative or management personnel in healthcare, community group representatives, technology designers, technology manufactures, health care and technology companies, marketing agencies, knowledge translation specialists, insurance bodies, regulatory agencies, political activists and decision-makers, and policy makers [22–25]. Some efforts have been made to understand barriers to the adoption of *specific* rehabilitation technologies using multi-disciplinary perspectives. For example, several published studies have explored the perspectives of individuals with disability, carers, *and* healthcare professionals on the adoption of health technologies like eRehabilitation programs [26, 27] and wearable functional electrical stimulation garments [28]. The perspectives of engineers, technology sellers and retailers, and academic researchers have also been considered in developing a model of functional electrical stimulation use over time [29]. However, arguably, further in-depth qualitative exploration of the factors that influence the success of rehabilitation technologies *more generally* is needed from a broader multi-disciplinary stakeholder perspective than is currently available. Therefore, the aim of this work was to develop a multi-stakeholder perspective on what influences the adoption of rehabilitation technologies.

## Methods

We conducted this study as part of a larger research project aiming to facilitate the co-design of a novel neurorestorative technology-based system for spinal cord injury (SCI). The system prototype is comprised of technology not currently readily accessible or used in clinical rehabilitation for individuals with SCI (e.g., brain computer interface, virtual reality). Focus groups were conducted as part of a day-long SCI rehabilitation symposium in June 2021 in Queensland, Australia. For context, morning symposium presentations focused on the current trajectory of SCI rehabilitation and novel neurorehabilitation technologies for SCI and were followed by a live demonstration of the system prototype. In the afternoon, symposium attendees were invited to participate in the focus groups. An exploration of the factors that could influence technology uptake was undertaken in focus groups, with the intention of applying these learnings to prototype development to maximise the likelihood of successful implementation in rehabilitation settings. All research was conducted with ethical approval obtained from the Griffith University Human Research Ethics Committee (reference number: 2019/958), with informed consent provided when participants registered for the symposium.

### Participants

Targeted recruitment was used for symposium attendees, to cover a wide variety of stakeholders in SCI rehabilitation who were both internal and external to the project. Targeted stakeholder groups included medical, allied health, human movement science, biomedicine, engineering, computer science, business, product development, design, marketing, funding, and lived experience of spinal cord injury. Attendees were identified through the project team’s professional networks and invited to the symposium through targeted emails. All symposium attendees were invited to participate in the focus groups. Pre-registration was available for the symposium, with attendees indicating their mode of attendance (i.e., in-person or virtual) and interest in participating in focus groups.

### Data collection

Focus groups were facilitated by individuals external to the larger research project, and who did not have technical knowledge of the prototyped technology. Participants were assigned to focus groups by facilitators using stratified randomisation across disciplines with the intention of creating groups whose participants spanned across multiple disciplines. A semi-structured focus group guide was used to cover twelve key topics related to the clinical uptake of rehabilitation technology: positive functional outcomes, cost, usability, availability, trust, acceptability, engagement with technology, stakeholder involvement, translation issues, likelihood of recommending, utility, and desirable information (Appendix). Development of the focus group guide was predominantly informed by the non-adoption, abandonment, scale-up, spread, and sustainability (NASSS) framework and complexity assessment tool [30, 31]. The NASSS framework and assessment tool categorize complexities in healthcare technology innovation according to the illness or condition, the technology, the value proposition, the intended adopters of the technology, the organization(s) implementing the technology, the external context for innovation, and how each of these interact and emerge over time. Questions were phrased broadly to reflect complexities in the adoption of rehabilitation technologies generally, with only a few questions targeted towards the system prototype demonstrated during the symposium. Focus groups were audio-recorded and transcribed by a professional transcription service. All identifying information was removed from transcripts prior to analysis, including names and organisational affiliations, to ensure participants’ anonymity and privacy. Following de-identification, participants were only identifiable by their focus group (i.e., Groups 1–5).

### Data analysis

A hybrid deductive-inductive approach to thematic data analysis was employed using a five-phase approach (Appendix) [32–34] and facilitated by the software package NVivo (March 2020 release; [35]. Three authors, with expertise in exercise physiology (KC), rehabilitation engineering (CS), and psychology (JM), were involved in analysis. All three authors began analysis by listening to audio recordings and reading audio transcripts from all five focus groups to achieve familiarisation. In consultation with the other two members, one author (JM) then developed an initial thematic framework. Codes were first generated in alignment with the a priori focus group questions while also being allowed to emerge directly from the recurring issues, opinions, and experiences discussed by participants. All codes were subsequently sorted and grouped across focus group questions into broader, higher order categories to form the initial thematic framework. Transcripts from all five focus groups were systematically indexed to the framework. Using the thematic framework and learning gained through indexing, all three authors worked together to construct and synthesise data into a set of thematic charts. All three authors continued to work together to define concepts, map the range and nature of phenomena, and find associations between themes with the purpose of providing explanations for the factors found to influence technology adoption. The approach to data analysis utilised is recognised as facilitating inclusion of diverse teams of researchers [36], which was considered important given the multi-stakeholder focus in this study, and adds breadth to the findings presented [37, 38].

## Results

The symposium had 58 attendees across in-person (n = 45) and online (n = 13) modalities. Symposium attendees were members of the project team (n = 14), members of the project’s external steering committee (n = 11), or external to the project entirely (n = 33). The expertise of symposium attendees spanned 13 fields, with many attendees belonging to more than one discipline. Fields of expertise included medical (n = 4), allied health (n = 20), human movement science (n = 6), engineering (n = 12), computer science (n = 2), business (n = 11), product development (n = 10), design (n = 2), marketing (n = 2), funding (n = 9), ethics (n = 1), research development (n = 6) and lived experience of spinal cord injury (n = 1). Of symposium attendees, 43 joined focus groups in-person (n = 35) or online via Microsoft Teams (n = 8).

**Fig. 1:**
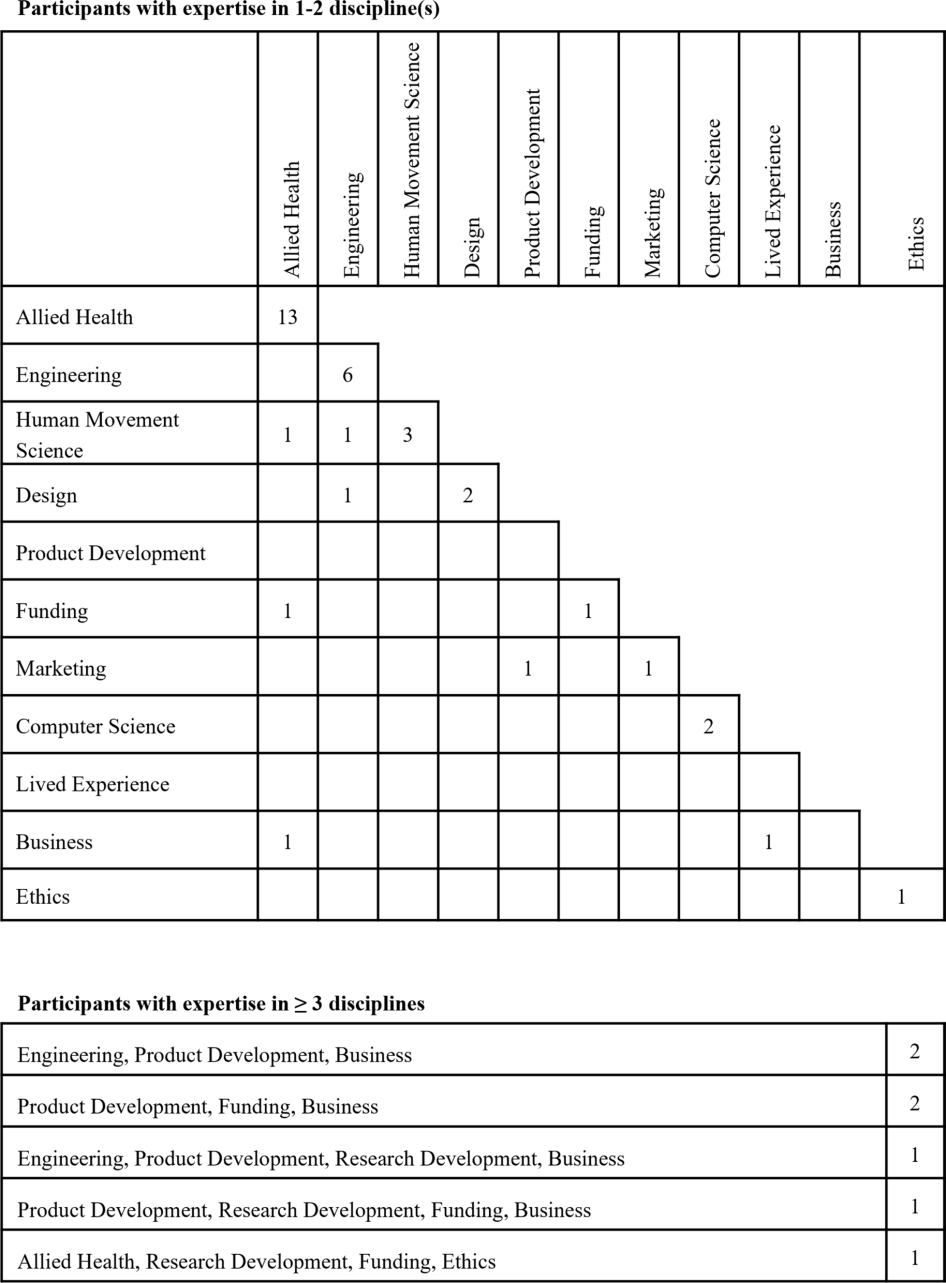
Focus group participants (n) mapped onto their area(s) of expertise. Participants with expertise in one to two disciplines and expertise in three or more disciplines are mapped separately

Of focus group participants, 12 were members of the project team, 8 were members of the project’s external steering committee, and 23 were external to the project entirely. Focus group participant expertise spanned 12 fields, with multiple participants belonging to more than one discipline (Fig. 1). Five focus groups were conducted simultaneously, with focus group sizes ranging from eight to ten participants. Groups 1 to 4 were in-person, and Group 5 was online.

Six main themes were identified in the analysis of focus group data: cost beyond the purchase price, benefits to *all* stakeholders, trust to be earned in technology, ease of technology operation, ability to access technology, and the ‘co’ in co-design. Main themes and sub-themes are described in text; see also Table 1.

**Table 1:** Main (bold) and sub-theme descriptions identified through analysis of focus group data

Main and sub-themes		Description
**1**	**Cost beyond the purchase price**	**Costs extend from the point of purchase through the lifespan of rehabilitation technology, are not only financial in nature, and are incurred across multiple stakeholder groups**
	Purchase and operational costs	Financial costs incurred in the initial purchase and then eventual replacement of technology as well as ongoing costs associated with technology operation, such as the purchase of accessories and consumables and maintenance fees
	Infrastructure requirements	Infrastructure requirements for the environment where technology will be kept and used, for example, physical space, noise levels, and internet connection
	Labour and time	Human labour and time necessary for technology set-up and use, including the number of people and the level of supervision required, and the ongoing commitment required from users to achieve desired outcomes
	Risk of harm to users	Potential harm to individuals resulting from technology use, for example, the risks to physical and psychological safety and the misuse of personal data
**2**	**Benefits to ‘all’ stakeholders**	**Desirable outcomes of rehabilitation technology are biopsychosocial in nature, contribute to the quality of life of individual users, and represent savings to the healthcare system**
	Physical function and health	Improvements in the physical function and health of technology users, that aid in the pursuit of their rehabilitation goals and reduce their incidence of re-hospitalisation
	Psychosocial wellbeing	Improvements in the psychosocial wellbeing of technology users, including feelings of empowerment and social connection resulting from and contributing to technology use
	Savings to the healthcare system	Savings to the healthcare system resulting from technology use, for example, due to reduced incidence of re-hospitalisation and increased efficiencies in service provision
**3**	**Trust to be earned in technology**	**Trust is not given easily and instead must be earned before stakeholders are willing to try, purchase, or recommend new technology**
	Supporting empirical evidence	Empirical evidence that demonstrates the benefits and safety of technology use from clinical trials with representative and diverse samples of potential users
	Transparency with stakeholders	Transparency with regards to possible benefits and risks of technology use, facilitated by the use of plain language for effective communication to potential users
	Trial availability before purchase	Ability to trial technology personally before purchase, commitment to ongoing use, or recommendation to others
	Recommendation by stakeholders	Recommendation or endorsement of trusted stakeholders, particularly peers who are experienced in technology operation
**4**	**Is it usable?**	**Rehabilitation technology that is easy to set up, use, and troubleshoot for those stakeholders who use technology**
	Intuitive operation	Technology that can be used intuitively with minimal effort; ideally “plug and play” technology
	Reliable operation	Technology that does not break down and can be used reliably by different users
	Customisability for individual users	Technology that is customisable to individual users without the need for unanticipated purchases or adaptions
	Instructional resources and technical support	Easy to understand, multi-format (e.g., audio-visual) instructional resources and readily accessible technical support
**5**	**Ability to access technology**	**Access to rehabilitation technology is influenced by its commercial availability, financial accessibility, and environment of use**
	Commercial availability	Technology that is available in commercial markets, particularly those that are local to payers
	Financial access	Technology that can be afforded by payers and that meets individual requirements for funding, for example, that has sufficient supporting empirical evidence
	Environment of technology use	Technology that is usable in a variety of environments to meet users’ individual needs and access preferences, including in hospitals, community settings, and users’ homes
**6**	**The ‘co’ in co-design**	**Rehabilitation technology that has been designed with stakeholders who will both use it and influence its use**
	Direct stakeholder engagement	Direct engagement of a diverse range of stakeholders, including people with disabilities, clinicians, technical experts, business stakeholders, funding bodies, and regulators
	Understanding of diverse needs and utilisation of expertise	Understanding of the diverse needs of stakeholders and utilisation of their expertise in the development of technology

### Theme 1: cost beyond the purchase price

Costs identified by participants extended from the point of purchase through the lifespan of rehabilitation technology, were not only financial, and were incurred across multiple stakeholder groups. Or, summarised by one participant, “cost and price are not exactly the same thing, in fact, they’re not the same thing” (Group 2). Costs were considered acceptable when technology meets the individual requirements of the stakeholder they are incurred by. That is, costs appeared to be acceptable when the specific cost(s) to each stakeholder were perceived to be offset by benefit(s) to them (see Theme 2 for benefits to individual stakeholders). Still, cost minimisation was supported amongst participants, with some discussion held around cost minimisation strategies. Cost–benefit analysis considerations and minimisation strategies varied both by the specific cost under consideration as well as the stakeholder they are incurred by, highlighting the importance of understanding the diverse needs of rehabilitation technology stakeholders (as outlined in Theme 6). As stated by one participant:

…it’s also like who benefits from a cost reduction, right? Because you can say okay… the cost is related to the patient, right? But it could also be to the organisation. Maybe you have a rehab gym that makes your whole organisation more efficient because you are able to treat more patients or because you’re able to reduce the number of hours and staff, so how to take that into account when it comes to the cost and the technology (Group 2).

In addition to the initial purchase cost and the cost of replacing rehabilitation technology, ongoing operational costs, such as licensing, accessories, consumables, and maintenance were discussed by participants. Strategies to minimise purchase cost included leasing, second-hand sale, separation of hardware and software, sale of individual components, and product tiers, while software updates were discussed as one approach to increase the lifespan of technology and minimise the cost of replacement. In terms of cost justification, it was suggested for example that “… you’ve got to look at the total cost of ownership as well because if these devices only last for 12 months and you’ve got to replace them on a schedule, then the cost can add up. Whereas, the exoskeleton hopefully would last the lifetime of the patient, so it might be worth 10 times the price” (Group 3). Infrastructure requirements for the environment where technology will be kept and used, including physical space, noise levels, and internet connectivity, were also referred to as costs by participants. Physical space was described as a commodity in rehabilitation settings, particularly where existing technology may have to be removed to make space for new technology. As a result, physical space requirements were described as being minimised by many participants through integration with technology already in use so that “ideally you could use that gear [equipment] with this rather than having to buy another one” (Group 4).

Human labour and time necessary for service providers to facilitate technology use and for individuals to be able to use technology were also considered costs of technology use. Examples described by participants included the number of people and the level of supervision required for technology set-up and use. These costs were described as being associated with inefficiencies for service providers and significant financial burden to individual users. From the perspective of service providers, a preference was widely expressed for “one-to-one or even one-to-multiple patients possibly” (Group 2). From the perspective of individual users, it was highlighted that it could cost “just for one hour [of therapy] up to $400-$500 because they’ve got to get transport, they’ve got to get the carer to come in, the carer doesn’t do anything for an hour or two, and then they’ve got to pay another $200-$300 to get them back home” (Group 2). Time required from individuals for technology use in the context of their other life occupations was also described as a cost, and that therefore “it’s got to be something you can incorporate into a routine that fits around somebody’s life” (Group 2).

Potential physical harm (e.g., “risk of fractures”, Group 3) and psychological harm (e.g., “distressed about seeing their legs move in a fake sort of, you know, in that virtual sort of environment [when they do not have that function]”, Group 1) were described as costs of using some types of technology. The misuse of personal data collected in the use of technology was also raised as a potential cost, with questions like “how much data is collected, where it’s collected, what types collected?” (Group 1) asked by participants. Concerns about the risk of harm were offset by transparency from technology developers, including transparent reporting of adverse events (e.g., following clinical trials), and the ability to trial technology with individual users before committing to ongoing use (see Theme 4 for ways trust is earned).

### Theme 2: benefits to *all* stakeholders

Outcomes of rehabilitation technology use described by participants were biopsychosocial in nature and were described as both contributing to the quality-of-life of individual users and representing savings to the healthcare system. Improvements in physical function and health were perceived as having the potential to improve the quality-of-life of technology users. These physical outcomes were considered most beneficial when they contribute to rehabilitation goals “specific to the individual” (Group 1), resulting in increased independence in activities of daily living and participation in meaningful and enjoyable life occupations. As explained by one participant:

It may not be that a particular device says “I’m going to get that person walking” but it’s going to have evidence to improve cross-sectional mass of muscles or cardiovascular fitness which in turn is going to lead to improved participation (Group 2).

Improvements in psychosocial wellbeing, including social connection and participation, were also described as having the potential to improve the quality-of-life of technology users. For example, rehabilitation technology was perceived as having the potential to improve users’ adjustment following an injury (Group 1), sense of “purpose” (Group 4), “feeling of control” (Group 3), and sense of “self-efficacy” (Group 5). Further, rehabilitation was described as “not only [about] being independent in their tasks of daily living in self-care but being able to find some activities that are meaningful to them and they get personal enjoyment from” (Group 2). The relationship between physical and psychosocial benefits appeared to be reciprocal, in that psychosocial benefits were described as including increased engagement with rehabilitation technology, which over the long-term was perceived to result in improved physical function and health, which was also described as increasing users’ psychosocial wellness. The integration of competition and gamification were described as ways to prevent technology use from becoming “stale”, create social connections, and allow users to have “fun” (Group 5), increasing user engagement and wellbeing (Group 3).

Improvements in physical, psychological, and social wellbeing were perceived as desirable not only to individual technology users but also to health service providers and insurance bodies (see Theme 6 for the importance of understanding the diverse needs of rehabilitation technology stakeholders). For these stakeholders, biopsychosocial benefits to technology users were perceived as having the potential to reduce reliance on the healthcare system over the long term by shortening hospital stays and reducing re-hospitalisation rates. Versatile technology that is “effective for 90% of the population” (Group 3) and that clinicians can “put on anyone and it goes” (Group 2) was also described as beneficial to health service providers. More specifically, versatile technology was linked to reductions in the number of hours and staff required to provide rehabilitation services and increased capacity to treat more patients, resulting in notable healthcare savings.

### Theme 3: trust to be earned in technology

Participants described trust as being not easily given but rather earned before stakeholders are willing to try, purchase, or recommend new rehabilitation technology. One way to earn trust appeared to be through robust empirical evidence supporting claims about the benefits of technology use (see Theme 2 for desirable biopsychosocial benefits to technology users) and harm minimisation to users (see Theme 1 for risks of harm to technology users). As stated by one participant: “there’s trust that the device is going to do them no harm and trust that this is going to improve their life somehow, I guess are the two things that I see” (Group 4). In the case of some potential payers, it was noted that minimum requirements for supporting evidence exist, for example:

What we are able to fund from a national [perspective], under the [insurance scheme] is covered under the legislation, it does have that exclusion around experimental treatment and then there’s also that consideration that we consider for necessary and reasonable about, and evidence base, and evidence based is generally level II [randomised, controlled trial] and level I [systematic review of level II studies] evidence (Group 5).

Clinical trials were described as needing to have representative and diverse samples of potential users, with exclusion criteria minimised to support understanding of the suitability and effectiveness of new technology for a range of users (see Theme 2 for the benefit of versatile technology in clinical settings). As expressed by one participant, “if the evidence that is emerging is coming out is very specific to a very small cohort, I’m not going to trust that then applies to all my patients” (Group 2). Participants indicated that such evidence needs to be transparently communicated to rehabilitation stakeholders so they can make informed decisions about technology use and establish realistic expectations about possible benefits. This kind of transparency was described as being facilitated by the use of plain language for effective communication to potential technology users. As said by one participant:

Being really truthful and I guess very clear about the mechanism of how this is going to work, setting expectations just so that people can make really good choices about their treatment and about their time, and also so that clinicians then understand how it works and they can also add to that health literacy as well (Group 5).

Still, stakeholders were portrayed as wanting to have the opportunity to trial new technology themselves before purchasing it or committing to its long-term use, particularly for “a massive piece of equipment” (Group 2). Trialling technology was described as important to grow users’ confidence in using technology and managing potential risks to their physical safety. Recommendation from trusted stakeholders was described as also influencing subsequent trust in or willingness to try new technology. However, importantly, the trust placed in recommendations varied by the stakeholders making and receiving them. For example, awareness and subsequent adoption of rehabilitation technology among individual users were described as being supported by recommendations from clinicians but also other users, who have a better understanding of the “intricacies” of day-to-day use of rehabilitation technology (Group 1; see Theme 6 for the importance of understanding the diverse needs of rehabilitation technology stakeholders). To this end, it was suggested that new technology is “going to need champions, I guess, isn’t it? Like trusted champions with different [stakeholder] groups, that’s different people presumably” (Group 4).

### Theme 4: is it usable?

The ease of setting up, using, and troubleshooting issues with rehabilitation technology were consistently described by participants as fundamental to adoption, with one participant stating that “a lot of good stuff goes by the wayside just because it isn’t inviting and easy to use” (Group 5). Usability was described as being supported by technology that can be used intuitively with minimal effort, to the extent that it is considered “plug and play” (Group 1) or “foolproof” (Group 3). For technology users, it was suggested that “you want it as the least amount of complexity in using it and understanding it as possible” (Group 1), particularly when “the person’s taking it home” (Group 2) and for older users who may not be “digital natives” (Group 1). Similarly, technology that can be used intuitively by clinicians was also described as valuable. Discussion about the costs of sending clinicians for “professional development to take a couple of days or a week to learn to use it to apply it to clients” (Group 2; see Theme 1 for discussion of the human labour and time costs associated with rehabilitation technology), was accompanied by concern about the dilution of knowledge passed between clinicians in high-turnover environments. As a result, some participants suggested that “it has to be something that a clinician with very little training can just pick up, figure out how to use it” (Group 2). Usability also appeared to be linked to reliability, with reliability used by participants to refer to technology that “doesn’t break down” (Group 5) and that also operates consistently across users. For example, questions such as “what happens the day that you press go and nothing happens?” (Group 3) and “to identify what is going to [be] best for this, can we try it on other people, is it going to work in this and that environment?” (Group 1) were raised by participants (see Theme 1 for discussion of the human labour and time costs associated with rehabilitation technology).

The customisability of technology to individual users was discussed as one way to make technology easier to operate, more reliable, and more accessible. Participants described undertaking their own technology modifications using tools like 3D printing, because there is a “disconnect between the end-user and [the] person designing it, like they didn’t understand what the needs were when they made it” (Group 3; see Theme 6 for the importance of direct stakeholder engagement to understand the diverse needs of rehabilitation technology stakeholders). Notably, it was highlighted that the degree of customisation offered to individual users needed to be balanced with the versatility required in clinical settings. One suggestion was that this balance could be found through:

Software [that] is there to enable some degree of personalisation and customisation for that individual’s needs, and then the hardware is that versatility perhaps that you’re looking for in a clinical setting, so that the two of them work together and hopefully in more of an optimal way (Group 1).

Ease of technology operation was also described as being supported by multi-format instructional resources and readily available technical support. Participants emphasised the importance of instructional resources available in multiple formats, including the use of “pamphlets and flow diagrams” (Group 2), “comics” (Group 2), “video tutorials” (Group 2), and “apps” (Group 4), to suit multiple “learning mode(s)” (Group 2), levels of “health literacy” (Group 4), and disabilities (e.g., “visually impaired or low vision”, Group 1). Relatedly, as highlighted by one participant, “one thing that stops [technology users] is poor sales and support… because you get people that want to use this technology and then something breaks and they may well go backwards” (Group 5; see Theme 2 for discussion of the desirable biopsychosocial benefits of technology use). Access to both in-person technical support (e.g., “like somewhere accessible in the community”, Group 4) and virtually delivered technical support (e.g., “or even like a Teams where they can take over your screen and set it all up for you”, Group 4) was described as desirable.

### Theme 5: ability to access technology

The ability to access rehabilitation technology was described as being influenced by its commercial availability, financial accessibility, and environment of use. More specifically, access to rehabilitation technology was described as being reliant on ensuring that it overcomes challenges associated with developing and launching technological solutions so that they are commercially available. Or, as summarised by one participant, “well, where would you buy it?” (Group 2). Perceived barriers to commercial availability included securing sufficient funding for research and development, navigation of regulatory approval processes, awareness of intellectual property rights, and delivery timeframes (see Theme 6 for the importance of direct stakeholder engagement so that relevant expertise may be utilised to overcome barriers such as these). Challenges associated with commercialisation in smaller markets were also raised, specifically the need to move to larger markets in other countries to “scale-up” (Group 2), which was described as resulting in limited access to affordable rehabilitation technology for some users.

Access was also described as being reliant on finding a payer for rehabilitation technology, whether it be health service providers, insurance agencies on behalf of individual users, or individual users themselves (see Theme 1 for discussion of financial costs associated with purchasing rehabilitation technology). Considerations included the affordability of technology for different payers and whether technology met the payers’ individual requirements (see Theme 3 for discussion of the types of empirical evidence required). As highlighted by one participant, “cost-based analysis for a piece of technology in the hospital is a lot different if you’re asking a patient to pay for it through their [public national insurance scheme] funding for example or personally” (Group 1). Access to rehabilitation technology was also discussed in relation to the environment it could be used in. Technology that is usable in a variety of environments was described as more accessible to potential users (see Theme 1 for discussion of how costs of rehabilitation technology adoption can be influenced by the environment of use). Examples of desirable environments included “I like people to have access to technology within their own home and own setting” (Group 4), “I want to see it in [rehabilitation gyms]” (Group 4), and “like somewhere accessible in the community that’s easy to get to” (Group 4). These options were considered necessary to meet the preferences of individual users. As summarised by one participant:

They’re looking at stuff close to home, transport’s an issue, cost of that is an issue, so it’s got to be something you can incorporate into a routine that fits around somebody’s life, what they’re doing that’s affordable. And I guess you’ll do that for a while, I imagine, after you’re newly injured, and you’re home and you’re doing rehab, but there’s a time where other stuff becomes a priority and do you keep on doing that? (Group 2).

Financial accessibility and environment of use appeared to be linked, in that the lower the purchase price of technology, the more environments it can be made available to use in (see Theme 6 for the importance of understanding the diverse needs of rehabilitation stakeholders). For example, one participant stated:

I guess you also have the issue of if it costs a lot, it’s more likely to be one thing that’s centred somewhere versus something that doesn’t cost quite so much that can be distributed to like a physiotherapy clinic rather than a spinal unit (Group 5).

### Theme 6: the ‘co’ in co-design

Participants involved in focus groups discussed a wide range of factors that they perceived to influence the adoption of rehabilitation technologies. These factors were presented from both their own perspective and on behalf of other stakeholders. However, participants also expressed the need to directly engage a diverse range of stakeholders in designing rehabilitation technology including people with disabilities, clinicians, technical experts, business stakeholders, funding bodies, and regulators. Some participants also discussed the value of including “boundary spanners” (Group 1), or “people who speak a bit of each language and sort of get each other to understand that we’re actually all saying the same thing or at least translate what the clinician is telling the engineer and so that we actually understand each other a bit better” (Group 1). Each of these stakeholders was described as adding value to rehabilitation technology development through their experience and expertise. However, particular emphasis was placed on the engagement of people with disabilities in the development of rehabilitation technology. For example, while participants relayed information gathered from people with disability through their work, several made a point to note that “I’m speaking on behalf of lived experience and I don’t personally have an impairment, so I don’t want to assume knowledge of everyone’s experience” (Group 1).

Stakeholder engagement was encouraged so that developers may better understand the diverse needs of each stakeholder group and utilise their expertise in the development of technological solutions. According to participants, not only do the needs and requirements of each stakeholder group vary but so does the threshold for technology adoption within each stakeholder group (see in particular discussions about varying thresholds for cost–benefit analyses in Themes 1 and 2, varying requirements for trust in Theme 3, and the importance of usability for both clinical and non-clinical users in Theme 4). As summarised by one participant, for example:

The end user is the primary person, then you have an operator of the technology, then you have someone who is purchasing it. So, you’ve got those three stakeholders involved and you have to take them all into account, but at the end of the day if you can, say, put a cost, I know it’s cold, but if you can say, okay, the cost-benefit says this to the purchaser, you’ve made that product feasible for that person. That doesn’t mean it’s feasible in terms of operational for the user, but it satisfies that stakeholder. But yeah, it’s a real wrestling match with those three (Group 1).

Particular importance was placed on the involvement of people with disabilities and clinicians at the need identification and verification stages of development. As highlighted by one participant:

…if you’re trying to make people walk again but they’d be more than happy just to be able to use their own bowel properly, define the problem first and get your end-users in to help you define the problem you’re going to solve (Group 3).

This was further emphasised by another participant who stated:

I would say what normally what really annoys us in our clinical team is that we don’t get kind of consulted at the top… they [developers] come to us with an already kind of a solution or a research idea which we don’t think is a problem or our clients don’t think is a problem (Group 2).

The inclusion of funding and regulatory bodies in the development of rehabilitation technology was also described as important to ensure technology met their respective needs and to increase the efficiency of the development process. An example provided by one participant was that “having advice at the beginning with device regulatory procedures” can help developers to know if they have “a much better and quicker way to market” because, e.g., “80% of your device has already been approved in another form in another device”, and therefore “save you finding there’s a roadblock just before you get there” (Group 4).

## Discussion

**Fig. 2:**
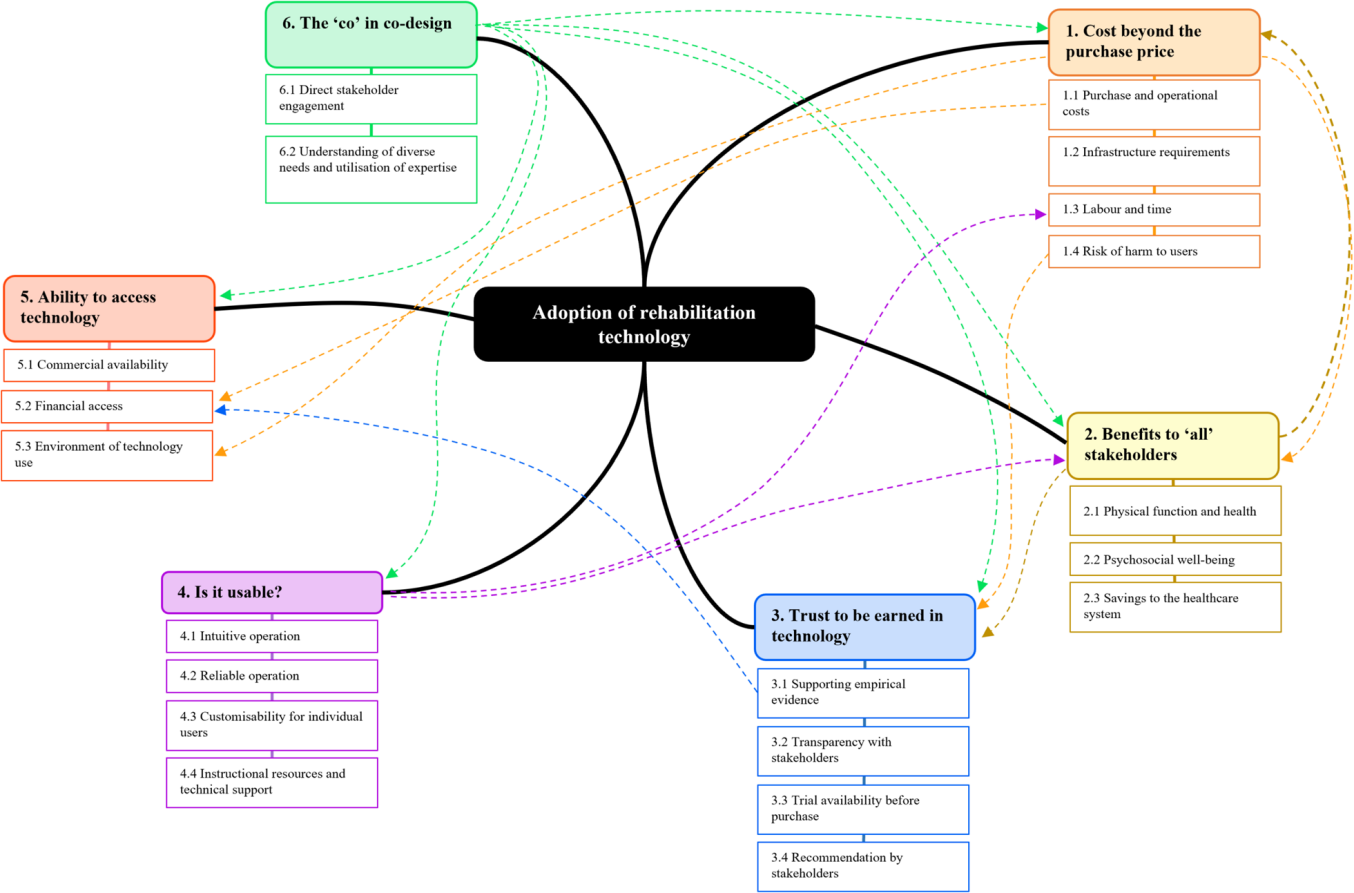
Map of relationships between main and sub-themes identified through analysis of focus group data. Bold lines denote main themes influencing the adoption of rehabilitation technologies. Coloured dashed lines denote relationships between main and sub-themes

Through qualitative analysis of opinions captured from people with disability, allied health, human movement science, computer science, design, engineering, ethics, funding, marketing, business, product development, and research development stakeholders, we identified six main themes influencing the adoption of technology in rehabilitation. The financial and non-financial costs of technology use, the potential for multi-stakeholder benefit from technology use, trust in technology, technology usability, and ability to access technology were all identified as being interrelated; with technology costs weighed in the context of their benefits (and vice versa), trust used to both reduce concerns about technology costs and increase belief in technology benefits, usability influencing technology costs (when poor) and enhancing technology benefits (when high), and access to technology influenced by factors like cost and the availability of supporting evidence. Perhaps most importantly, these five themes were connected to the sixth theme– the ‘co’ in co-design—which highlighted the importance of direct stakeholder engagement in the development of rehabilitation technologies to better understand stakeholders’ diverse needs and utilise their expertise in development (Fig. 2). We discuss each of these themes, including their relationships to one another, in the context of other work in the fields of technology and rehabilitation.

### Financial and non-financial costs as barriers to technology adoption

Participants conveyed that “cost and price are not exactly the same thing” (Group 2); that is, that there are many other costs beyond device-related purchase, maintenance, and eventual replacement relevant to the acquisition and ongoing use of a device. This is consistent with existing literature that identifies cost considerations including infrastructure requirements such as physical space [19, 26] and internet connectivity [26, 27]; clinical labour- and time-related requirements such as adequate staffing [19, 39] and fit with within clinical workflow [18, 40, 41]; and considerations for individuals with disability to access and use devices, including travel to (specialist centres) where they are located [29], competing life demands [29, 42], and energy constraints [43]. When not considered, these costs can result in clinicians refraining from adopting new technology if they believe that it has the potential to increase their workload [40] and individuals with disability finding the “hassle” and “effort” of accessing and using technologies outweigh the benefits [44]; this is particularly the case when technical issues reduce usability [26]. Further, at least until rehabilitation technologies are more widely accepted, they will likely not replace traditional rehabilitation [26] and, thus, create additional time demands—rather than decreasing them—for individuals engaging in rehabilitation. These financial and non-financial implications tend to be incurred mostly by both users with disability and health service providers and, therefore, their perspectives are particularly important to consider in the development of rehabilitation technologies.

Further, similar to other work, our findings indicate that serious consideration must also be given to the risk of psychological and physical harm [28, 29, 40, 45] and concerns about data privacy and security [23, 26, 40]. When not appropriately addressed, for example through clinical trials, these are significant barriers to the regulatory approval of technology, delivery by health service providers, and use among individuals with disability. Of importance, when balanced by technology benefits, technology costs were considered to be acceptable to participants. As such, technology users may accept inconveniences associated with technology providing it yields benefits [29, 42].

### Desirable benefits of technology use and how they vary by stakeholder

Desirable benefits to technology users with disability were biopsychosocial in nature, aligning with previous literature that highlights the importance of considering a wide range of physiological, psychological, and social benefits of technology use during its development [39, 45–48]. This includes, for example, recovering sense of agency, providing opportunities for participation, and positive effects on users’ self-image [48]. We further found suggestion of a relationship between user enjoyment with motivation to use technology [49], likely contributing to longer-term adoption. Other desirable benefits of rehabilitation technology were related to savings to the healthcare system and efficiencies in service provision. Other work has also indicated that technology has the potential to increase competitiveness in clinical settings through time and cost efficiencies, leading to quicker access to care for individuals with disability [40] and reduced clinical workload [6]. However, usability issues can instead turn this potential benefit into a cost, with new technologies instead creating more work for clinicians [40]. Relatedly, the appropriateness of technology for only a small subsample of individuals with a specific condition or injury has previously been identified as a possible barrier to the widespread adoption of particular rehabilitation technologies [42], particularly in instances where technology is seen as inappropriate for a large percentage of clients [41]. It is therefore important to understand health professionals’ clinical reasoning processes as a means of informing technology development to help “match” technology to the setting in which it will be used, including the demands of clinicians [50].

### The importance of trust in technology and how to earn it

Trust was important to a range of rehabilitation stakeholders, particularly users with disability, clinicians, and funding bodies, so that stakeholders can make informed decisions and establish realistic expectations about potential benefits of adopting technology. Indeed, assistive technology use is more than three times higher among individuals who perceive technology to be important to their rehabilitation [10], and clinicians are more likely to use a tool when they understand its benefits and refrain from using tools if they are sceptical about the value they bring to their clinical practice [40]. However, the expectations of rehabilitation technology users have been reported to be unduly high [29]. This is of particular concern as expectations about the benefits of rehabilitation technology have been linked to compliance among users, with compliance varying as a result of whether or not expectations regarding technology use are met [29]. In addition to rigorous empirical evidence, trust was discussed in focus groups as being facilitated through technology use or, more specifically, the ability to trial new technology. Self-autonomous research [29, 51] and trailing new technology [51] are ways that individuals with disability gather information about new assistive technology (including information about potential benefits) and address concerns about potential risks associated with technology use. Further highlighting the importance of engaging a range of stakeholder in the development of rehabilitation technologies, recommendation from “trusted champions” (Group 4) was reported by participants to encourage technology adoption [28, 50], as was peer influence, leadership endorsement, and institutional support in clinical settings [40].

### Technology usability can either facilitate use or increase associated costs

Usability was identified as having the potential to facilitate technology use but also negatively impact its adoption. Focus group participants raised usability considerations consistent with the existing literature including: need for user-friendly and intuitive operation by individuals with disability and clinicians with varying levels of experience and exposure to technology [26, 40, 41]; insufficient training, lack of time to learn new technology, resourcing requirements for sustaining training programs, and training programs that focus on technical knowledge without considering workflow changes associated with technology use [40]; and reliability and technical issues resulting in frustration to users and interruptions to the provision of clinical care [49]. Therefore, it is important that not only is technology easy to operate for a wide range of potential users, but that appropriate training and support resources are provided to improve ease-of-use, and that clear expectations are set about device usability (i.e., who will be able to use technology independently).

Technology usability is perhaps best facilitated through detailed understanding of the diverse needs of the user, based on utilisation of expertise through stakeholder engagement. As in other work [27], technology customisability or personalisation was identified as one way to improve technology usability. However, balance must be found between customisability options and avoiding overwhelming clinicians with an excess of decision-making requirements [39], which reduces usability. Ongoing support to use technology is also considered desirable, as lack of technical resources and support have been widely linked to technology underutilisation and abandonment [27, 28, 39, 52]. To support the individual needs to relevant stakeholders, focus group participants emphasised the importance of multi-format instructional resources (i.e., written, visual, auditory), and technical support available via both in-person and virtual mediums. Other work suggests that there are significant differences in technology use and abandonment between individuals who are actively receiving rehabilitation services and those who are not, with clinician support positively influencing technology utilisation [10].

### Technology can be difficult to access for several reasons

Inability or difficulty in accessing rehabilitation technology was described as a significant barrier to its adoption. Lack of awareness of currently available technologies or their full range of applications hinders adoption, with active promotion of technologies and their benefits encouraging use [39, 40, 42]. Unsurprisingly, access to technology was related to the theme “cost beyond the purchase price”, specifically financial access and the influence of the environment on technology access were both related to cost. The inability to afford rehabilitation technology and, relatedly, refusal of funders to pay for the financial costs associated with acquiring or using rehabilitation technology is a notable barrier to access [26, 28, 42]. Notably, factors like the availability of rigorous empirical evidence to support technology use and address safety concerns, were reported by focus group participants to influence funders’ willingness to pay for financial costs. The environment in which technology can be used also has the potential to limit access to it; with technology that is usable in a variety of environments described as more accessible. Technology costs varied by environment of use with additional costs associated with travel, support staff, and time dedicated to other occupations likely to be incurred, which may limit adoption [26]. Cost was also identified as influencing the environments in which technology is available, with an inverse relationship between the purchase price of technology and the number of environments it can be made available in. However, we note that environment of use has different connotations depending on the technology under examination. For example, following a systematic review, Jacob, Sanchez-Vazquez [40] reported that the mobility of mHealth tools accessed via mobile phones is mostly seen as a facilitator of use, with value placed on portability of tools enabling clinicians to access information and complete tasks anytime and anywhere. Therefore, access to rehabilitation technology is often complex and best addressed through active engagement of rehabilitation stakeholders during technology development.

### The co-design of technology by rehabilitation stakeholders

Focus group participants consistently expressed the need to directly engage a diverse range of stakeholders in designing rehabilitation technology. Clinicians, technical experts, business stakeholders, funding bodies, regulators, and “boundary spanners” (i.e., stakeholders who transverse stakeholder groups) were among the stakeholders described as adding value to rehabilitation technology development through their experience and expertise. Particular emphasis was, however, placed on the engagement of people with disabilities in the development of rehabilitation technology. Stakeholder engagement was encouraged so that developers may better understand the diverse needs of each stakeholder group. With regards to individuals with disability specifically, our and other work has highlighted how intraindividual variation in needs, requirements, and threshold for technology adoption can influence adoption of rehabilitation technologies. For example, acceptance of disability changes over time and has the potential to influence willingness to engage in rehabilitation activities [29, 44]. Some work suggests that although there appears to be considerable overlap in factors important to individuals with disability and healthcare providers, individuals with disability and their formal and informal caregivers give more emphasis to factors related to the individual while healthcare professionals emphasise the importance of factors related to their organisational context [26]. Of further consideration is that benefits to one stakeholder group may inadvertently disadvantage another. For example, innovations primarily oriented towards readily-commercialisable technologies or to the interests of investors may not satisfy health system or technology users, and technology developers without healthcare knowledge may overestimate the value of their technology or make unfounded assumptions on behalf of technology users [25]. Notably, in focus groups, utilisation of the experience and expertise of a wide range of rehabilitation stakeholders was described as contributing to efficiencies in the technology development process and therefore a quicker path to market with technology that meets stakeholder needs. As a result, it is important to involve a range of stakeholders throughout the technology lifecycle, not only during the initial and developmental stages but through to end-product testing [53], the generation of user-tailored operational manuals and clinical training resources [50], and even service delivery [52] and technology promotion.

Our findings about co-design reinforce what is already widely recognised: technology users with disability continue to remain excluded from the development of rehabilitation technology, which ultimately leads to higher rates of technology abandonment. Engaging users in the development, planning, and implementation of rehabilitation technology is likely to positively influence its adoption. Our findings about co-design also reinforce the need to engage a wider cohort of rehabilitation stakeholders in the development of technology, beyond even those who participated in the focus groups.

### Implications

Our work is unique in the wide multi-disciplinary approach taken to both collecting and analysing data. The 43 participants engaged in focus groups spanned over 12 rehabilitation fields that influence the supply and demand of rehabilitation technologies, 74% of whom were not individuals with disability or clinicians, with purposeful distribution of these fields across the five focus groups. Further, data analysis was undertaken by a multi-disciplinary stakeholder team with clinical and engineering backgrounds. Our findings therefore emphasise the value of developing a broad multi-disciplinary perspective of factors that influence the adoption of rehabilitation technologies and the willingness of these stakeholders to contribute their relative expertise. Many of the factors that contribute to the underutilisation and abandonment of rehabilitation technologies may be addressed by utilising the experience and expertise of stakeholders who influence its supply *and* demand.

## Limitations

Although focus group participants spanned a wide range of rehabilitation fields, some fields were underrepresented. Only a small number of individuals with a disability participated in focus groups, and there was no representation of medical doctors or stakeholders with regulatory and policy expertise. This was due to participant availability and conflicts with participants’ other personal and professional responsibilities e.g., individuals with lived experience of a spinal cord injury may have occupations outside of the technology design and development field limiting their ability to attend a full day symposium. Further, while stakeholders were purposefully distributed across focus groups, there was insufficient attendance for one representative from each stakeholder group to participate in all focus groups. Therefore, future work would be well-guided to compare the perspectives from our focus groups with those from a broader sample of individuals with lived experience of disability, medical expertise, and regulatory and policy influence.

Also of consideration is the context within which focus groups were conducted, and the contexts within which the specific stakeholders involved in focus groups typically operate. For example, perspectives expressed in focus groups were likely anchored by the neurorestorative technology-based system demonstrated during the symposium, including the specific technologies (e.g., functional electrical stimulation, brain-computer interface, virtual reality) and the intended population of use (SCI). For example, some of the other potential benefits of rehabilitation technologies not raised in focus groups include enhanced patient-clinician communication [40] and the ability to participate in rehabilitation activities outside of clinical treatment hours [26]. We further note that participants in our focus groups were representative of rehabilitation stakeholders within Australia and that, therefore, the perspectives they expressed are reflective of cultural biases within Australia and of the Australian healthcare system. Upon completing a review of mobility-assistive technology literature, Alqahtani et al. [18] concluded that further research is needed on a global level to determine the development needs and priorities of technology users as these vary between regions even within countries. The authors noted that future areas of research and development were mostly identified using the voices of users from high-income countries, indicating a lack of research investigating users’ opinions in low-income countries.

## Conclusions

In our work we present a multi-disciplinary perspective on factors that facilitate the use of rehabilitation technologies and lead to their underutilisation and abandonment, as well as relationships between them. Of particular importance when considering the adoption of rehabilitation technology is the perceived benefits of adoption needing to outweigh the costs incurred, the need for developers to earn the trust of other rehabilitation stakeholders, and the need for technology to be easy to use and readily accessible. Further work is needed to determine the optimal balance between each of these factors for relevant stakeholder groups; for example, which benefits are necessary for each cost associated with use to be considered acceptable. Therefore, meaningful engagement of a wide range of the stakeholders who influence the supply and demand of rehabilitation technologies is recommended when developing and delivering them.

## Data Availability

The dataset generated and/or analysed during the current study are available from the corresponding author on reasonable request.

## Appendix

### Semi-structured focus group guide

**Table:**

Theme	Question(s)
Positive functional outcomes	The research project aims to develop a rehabilitation technology to improve outcomes for individuals with spinal cord injuries. From your perspective what would constitute a positive functional outcome for individuals with SCI?
	In your view, what are other outcomes that could be classified as positive indicators for individuals with SCI?
	Participation in and use of rehabilitation technology and interventions can be promoted to patients at multiple levels (from shortly after injury in a hospital setting, to multiple years after injury at home). In your field or position, what is required to support the use or promotion of technology? What do you support or base these recommendations on?
	The research project has the capacity to collect and analyse large amounts of data. What data would be of relevance to you and why?
Cost	Cost is an important consideration for technology in rehabilitation. From your perspective what are the key considerations that need to be addressed relating to the cost of technology?
	The cost of technology is distributed across a spectrum from low to high cost. What would justify an elevated cost of technology (for example providing someone with a low-cost phone application versus the costs associated with robotics)? How would this justification be demonstrated?
	What information do you require to demonstrate that a rehabilitation technology or intervention is value for money?
Usability	The usability of technology is proposed to influence its adoption. What elements make a piece of technology more or less usable (or easier or more difficult to use) than another? What are some non-negotiables for the usability of technology? Are there restrictions relating to the usability of technology that are non-negotiable?
	Can you detail any alterations you have previously made or know others have made to available technologies to increase its usability for individuals with SCI?
Availability	From your perspective, what are the key hurdles that need to be addressed regarding the availability of technology for the purposes of rehabilitation?
Trust	Trust in the developed technology is proposed to be an important component of adoption. How do you define trust in technology? What is the required threshold to establish trust?
	What obstacles need to be addressed in order to increase trust in technology?
	Do the levels of required trust in technology change depending on the technology being used (e.g., the trust required to use a prosthesis versus assisted cycling technology?) What factors influence the level of trust required?
Acceptability	Acceptability of the technology is another important proposed consideration for adoption. What elements do you think need to be included in a device to improve its acceptability (for example its compatibility with existing technology, practices or lifestyles, functionality and likeability) for individuals with SCI?
Engagement with technology	Usability, availability, trust and acceptability are proposed to be important considerations for engagement with technology. In your experience what are some of the main issues that restrict individual’s engagement in technology-based rehabilitation interventions? What strategies have you used in the past to increase user engagement?
Stakeholder involvement	Considering the technology development cycle, what level of involvement do you and your stakeholder Group need to be involved? At what points, regarding which aspects, how deeply?
	The activities of the translation Group centre on the idea that the development of technology with the best potential for translation requires the input of as many stakeholders as possible throughout all stages of the development process. Does this approach resonate with you? Do you think this approach is feasible? Why or why not?
Additional translation issues	What additional issues do you think restrict the translation of technology from a research setting to a “real world” setting?
	What are the biggest challenges associated with the implementation of technology for rehabilitation?
Likelihood of recommending	This project relates to the development of the prototyped technology. Based on your understanding of this technology would you recommend its use for individuals with SCI? Why or why not?
	What benefits do you think this technology could provide the users, such as individuals with SCI and healthcare workers?
Utility	What modifications do you think are required to increase the utility of the prototyped technology for individuals with SCI?
Additional information	What additional information regarding this technology would you like to have?

### Steps undertaken during qualitative data analysis

**Table:**

Phase	Detailed description of steps taken
Familiarisation	KC, CS, and JM achieved familiarity by listening to audio recordings and reading transcripts independently
	KC, CS, and JM met to discuss their initial ideas and recurrent themes
Thematic framework development	JM analysed the transcripts independently to generate an initial list of codes on the basis of the a *priori* focus group questions and study aims as well as recurring issues, opinions, and experiences discussed by stakeholders
	JM sorted and grouped the initial list of codes across focus group questions into broader, higher order categories, to form the initial thematic framework
	KC, CS, and JM met to review and refine the initial thematic framework, ensuring that there were no obvious areas of overlap or omission
	JM finalised the initial thematic framework, which included 100 descriptor codes organised into 15 categories. Each code and category was accompanied by a brief descriptor to enable consistent application across transcripts
Indexing	JM worked independently to systematically index transcripts from all five focus groups through application of the thematic framework
Charting	KC, CS, and JM worked together to identify relationships between categories in the in thematic framework and derive main and sub-themes, which were used to construct a set of thematic charts. Each main theme was charted separately
	JM worked to summarise the issues, opinions, and experiences discussed by stakeholders in the constructed thematic charts through abstraction and synthesis. Whole group analysis was conducted so that each row on thematic charts was designated for one of the five focus groups
Mapping and interpretation	JM worked together with KC and CS to define concepts, map the range and nature of phenomena, and find associations between themes with the purpose of providing explanations for the factors found to influence technology adoption

Adapted from approaches to qualitative analysis described by Pope et al. [34], Ritchie and Spencer [32], and Ritchie et al. [33].
